# Expertise differences in anticipatory judgements during a temporally and spatially occluded task

**DOI:** 10.1371/journal.pone.0171330

**Published:** 2017-02-07

**Authors:** Joe Causer, Nicholas J. Smeeton, A. Mark Williams

**Affiliations:** 1 Liverpool John Moores University, Research Institute for Sport and Exercise Sciences, Tom Reilly Building, Liverpool, United Kingdom; 2 University of Brighton, Centre for Sport and Exercise Science and Medicine, Welkin Laboratories, Eastbourne, Brighton, United Kingdom; 3 The University of Utah, Department of Health, Kinesiology, and Recreation, College of Health, Salt Lake City, UT, United States of America; Universidade de Tras-os-Montes e Alto Douro, PORTUGAL

## Abstract

There is contradictory evidence surrounding the role of critical cues in the successful anticipation of penalty kicks in soccer. In the current study, skilled and less-skilled soccer goalkeepers were required to anticipate when viewing penalty kicks that were both spatially (full body; hip region) and temporally (–160 ms, –80 ms before, foot–ball contact) occluded. The skilled group outperformed the less-skilled group in all conditions. Participants performed better in the full body condition when compared to hip region condition. Performance in the hip only condition was significantly better than chance for the skilled group across all occlusion conditions. However, the less-skilled group were no better than chance in the hip condition for the early occlusion points when predicting direction and height. Later temporal occlusion conditions were associated with increased performance both in the correct response and correct direction analyses, but not for correct height. These data suggest that postural information solely from the hip region may be used by skilled goalkeepers to make accurate predictions of penalty kick direction, however, information from other sources are needed in order to make predictions of height. Findings demonstrate how the importance of anticipation cues evolve over time, which has implications for the design of training programs to enhance perceptual-cognitive skill.

## Introduction

In sport, the inherent limitations in reaction time and movement time necessitates that athletes anticipate or predict future events based on limited preparatory information [1]. In order to effectively deal with such constraints, athletes possess a wide range of perceptual-cognitive skills, including the ability to: recognize advance (i.e., early arising) visual information (or cues); identify patterns/structure in play; and develop an awareness of likely event probabilities [2]. An expert athlete can limit the volume of information processed to generate a perceptual representation by selectively attending to more pertinent cues [3]. Furthermore, task-specific knowledge developed through experience is thought to help expert players look at these more important areas of the environment, using previous experiences to develop situational probabilities and allowing more effective processing of contextual information [4].

The availability of cues and time they become available within the display will directly influence a performer’s perceptual strategy and ultimately the accuracy of their judgements [5]. Traditionally, representative tasks paired with occlusion paradigms have been used to examine perceptual-cognitive skills. Temporal occlusion involves editing a film into specific time phases where progressively longer durations of a movement is presented. This paradigm has been frequently used to distinguish between skill levels in advance cue utilization [6]. Performance in later temporal occlusion conditions is generally reported to be significantly higher than earlier occlusion periods. This finding may be due to the availability of more important cues in the later stages of a movement, or it has been suggested that an increased viewing time in the later conditions allows the player to access more cues in the whole movement and not respond to just the cues near the end of the movement [7].

The temporal occlusion approach can only indicate the time frame that the cues are utilized, and not what cues are utilized [8, 9]. A complimentary approach is the spatial/event occlusion paradigm, which is a technique that masks an important area/cue in the visual field to examine how it affects cue utilization or information pick-up [10]. A decrease in performance suggests that the occluded area contains key information concerning that particular movement [5]. However, there is debate in the literature as to whether or not, in tasks such as the soccer penalty kick, goalkeepers focus on local postural cues (e.g. non-kicking foot), or they use a more global processing of cues and utilise a specific central visual fixation point (e.g. hips) to processes relative motion [11]. A combination of these temporal and spatial occlusion paradigms, to isolate specific regions, could help determine which of these techniques is being utilised by skilled individuals, and determine the relative contribution of specific postural cues.

In the soccer penalty kick, the limits of human reaction time [12] and the considerable temporal demands of the task [13, 14], necessitate that goalkeepers must anticipate ball direction before the taker strikes the ball. Therefore, researchers have endeavoured to determine skill-based differences in the use of visual cues emanating from the kicker. However, there has been considerable debate into the most effective areas to fixate gaze when attempting to anticipate a soccer penalty-kick.

Seminal research highlighted the importance of the angle run-up, the arc of the leg on approach to the ball, and angle of the kicking foot and hips prior to ball contact [15]. In contrast, follow-up studies revealed that expert goalkeepers spent a higher proportion of time fixating on both the non-kicking and kicking leg rather than the hips, while novices predominantly fixated on the, arms, trunk and hips [16, 17]. Kinematic analyses have cited the angle of the hips and kicking foot as reliable predictors of ball direction [18]. A more recent kinematic analysis has demonstrated that important predictive cues evolve over the movement and are all located in the lower part of the body [19]. Specifically, at 150 ms before foot-ball contact the non-kicking foot angle, the knee angle of the kicking leg, and the speed of the kicking foot are important. Whereas at ball contact, the kicking foot angle, the hip angle, and the movement direction of the kicking foot are more important.

Although there is contradictory evidence, there seems to be some consistency in regards to the view that the hip region provides valuable information. Moreover, players have verbally reported the orientation of the hips to be useful in determining penalty-kick direction [15]. Specifically, if the ball is directed to the goalkeepers left (assuming a right-footed penalty-taker), the hips slope away from the goalkeeper, whereas if the ball is travelling right the hips are square to the goal. Further support for the role of the hip region in penalty-kick anticipation was provided by Causer and Williams [20], who manipulated cues emanating from this region. Playing uniforms were developed that used patterns to disrupt the alignment of the hips. Performance significantly decreased in the experimental conditions where the hips were manipulated when compared to a control uniform. These data suggest that disrupting the pick-up of information from the hip region can be detrimental to anticipation performance, implying there is critical information emanating from this region.

However, it may not be the hips themselves where the critical information is derived, but rather it may be the relative motions or relationships between the hip region and other information sources. Some support for this notion have been provided by Piras and Vickers [11], who reported that goalkeepers utilize a ‘visual pivot’ strategy where point of gaze is centrally located mid-way between the ball and hip region in order to enable optimal use of the fovea and parafovea. Therefore, in order to determine the role of the hip region in making anticipation judgements in the penalty-kick, we use the spatial occlusion paradigm to remove information from all other regions other than the hips throughout the kick. By isolating this specific region, we can better identify how constraining access to certain information affects anticipation performance. The temporal occlusion paradigm will also be used to identify the time-course of the involvement of the hip region as a critical cue.

Our aim in the current study was to examine the effect of expertise on the accuracy of anticipatory judgments during temporally and spatially occluded penalty kicks. Using a highly skilled sample of goalkeepers, we use a novel spatial occlusion technique to isolate the role of specific cues in the successful anticipation of penalty kicks. It is hypothesized that there will be significant performance decrements for both groups in the spatially occluded conditions where only information from the hips is presented when compared to the full body condition [18, 21]. We also expect the skilled group will perform significantly better than the less-skilled group in all conditions [22]. Finally, we predict that both groups will perform significantly better in the later temporal occlusion conditions, compared to the earlier conditions [20, 23].

## Materials and methods

### Participants

Twenty-four male soccer goalkeepers volunteered to take part in the study. Participants were divided equally into either a skilled or less-skilled group based on playing level and experience. The skilled group consisted of 12 players (mean age = 26.8, *SD* = 4.3 years) who currently played professional or semi-professional soccer and had been participating in the sport for an average of 10.8 (*SD* = 3.4) seasons. The less-skilled group included 12 players (mean age = 25.8, *SD* = 5.2 years) who had only recreational playing experience. Participants provided written informed consent and were free to withdraw from testing at any stage and approval for the study was gained via the Liverpool John Moores University Research Ethics Committee.

### Test film

The test film was produced in conjunction with a professional soccer club in the UK. Four full-time, academy players were filmed from the goalkeeper’s perspective taking penalty-kicks. The film clips were recorded using a digital video camera (Canon DM-XM2 PAL, Tokyo, Japan) positioned in the middle of the goal at eye level (1.7 m). The players were asked to take the penalty-kick using the strategy that they would use in normal competition. Two of the players were right footed and two were left footed penalty takers. A regular dimension goal was used and players were required to shoot into each of the four corners of the goal in turn. A video camera was positioned behind the goal to ensure that each penalty crossed the goal line within 1 square meter of each corner. If the ball finished outside of these areas, the trial was discarded. The film clips included the penalty taker’s approach to the ball and all his preparatory actions until the ball was kicked. Players were required to place three penalties in each corner providing a total of 48 penalties.

**Fig 1:**
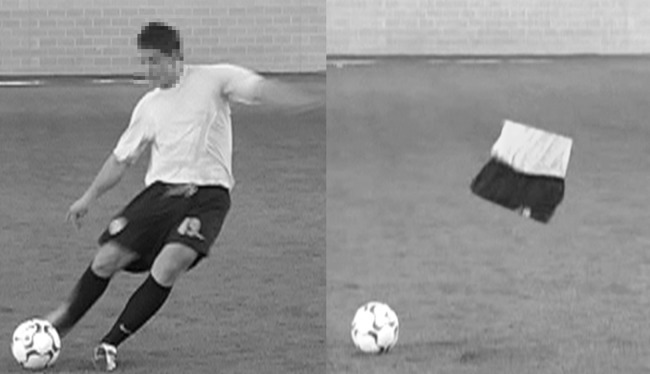
Ball contact for the full body condition (left) and hip region condition (right).

The footage was then digitally edited using Adobe Premiere Pro CS4 software (Adobe Systems Incorporated, San Jose, CA) so that each clip was temporally-occluded at either the moment of ball contact, 80 ms before ball contact (-80 ms) or 160 ms before ball contact (-160 ms). These occlusion points have been used successfully and shown differences in previous studies [23], allowing data to be accurately compared between studies. A second test film was created by spatially editing the temporally-occluded clips using Mokey 4.0 (Imagineer systems Ltd, NY) to remove all of the body segments of the player, apart from the hip region (see Fig 1). The background environment replaced the areas where the body segments had been deleted; the ball was left in the clip as a reference point.

### Procedure

The film clips were back projected, using a LCD video projector (Hitachi CP-X345, Yokohama, Japan) onto a 2.7 m x 3.6 m large projection screen (Draper Cinefold, Spiceland, IN). Participants stood 3.5 m away from the screen so that the film image subtended a visual angle of approximately 70° in the horizontal and 55° in the vertical direction; these angles and distances were used to match those of a penalty kick in an actual match setting. Participants were required to verbalize the direction of the penalty kick (top left, top right, bottom left, bottom right), as well as moving as if there were trying to save the penalty kick. The movement was not recorded as a dependent variable, but used to increase the fidelity of the task. Foam crash mats were located either side of the participants such that they could execute an actually dive as in the match situation. No feedback was given in relation to response accuracy. A sample of six random practice trials was shown pre-experiment for each test film to help the participants familiarize themselves with the task. After familiarization, the two test films (full body, hip region) were presented, with a 5-minute break in between. The order of the test films was counterbalanced across participants. Shot presentation sequence (penalty kick outcome and temporal occlusion condition) was randomized across the conditions. Each trial lasted approximately 4 seconds, with 5 seconds between trials for the participant to response and reset for the next trial.

### Statistical analysis

Correct response (%) was measured by comparing the participant’s response on a trial to the location the ball crossed the line. Successful performance was recorded when the participant correctly predicted both the direction and height of the penalty-kick. In addition, individual analyses of the percentage of penalties where the participants correctly anticipated the height of the penalty, and separately the direction of the penalty, were conducted. All dependent variables were analyzed using a 2 Group (skilled, less-skilled) x 3 Temporal Occlusion (-160 ms, -80 ms, ball contact) x 2 Spatial Occlusion (full body, hip region) mixed design ANOVA. Bonferroni-corrected independent t-tests were conducted to examine whether performance across skill groups and occlusion conditions differed from the 25% chance level. Effect sizes were calculated using partial eta squared values. Significant effects were followed up using bonferroni corrected pair-wise comparisons. The alpha level for significance was set at 0.05. The Greenhouse-Geisser correction was applied when violations to sphericity were observed.

## Results

### Correct response (%)

There was a significant main effect for group, *F*_1, 22_ = 290.59, *p* < 0.001, partial eta squared = 0.98. The skilled group recorded significantly higher accuracy scores (*M* = 70, *SD =* 14%) when compared to the less-skilled group (*M* = 45, *SD =* 12%). There was a significant main effect for temporal occlusion, *F*_2, 44_ = 209.56, *p* < 0.001, partial eta squared = 0.91. Response accuracy was significantly higher in the ball contact (*M* = 71, *SD =* 18%) condition when compared to the -80 ms (*M* = 56, *SD =* 14%; *p* < 0.001) and -160 ms (*M* = 46, *SD =* 14%; *p* < 0.001) conditions. Response accuracy was also significantly higher in the -80 ms, compared to the -160 ms condition (*p* < 0.001). There was a significant main effect for spatial occlusion, *F*_1, 22_ = 248.16, *p* < 0.001, partial eta squared = 0.92. Response accuracy was significantly higher in the full body condition (*M* = 63, *SD =* 17%; *p* < 0.001) compared to the hip region condition (*M* = 52, *SD =* 18%).

**Fig 2:**
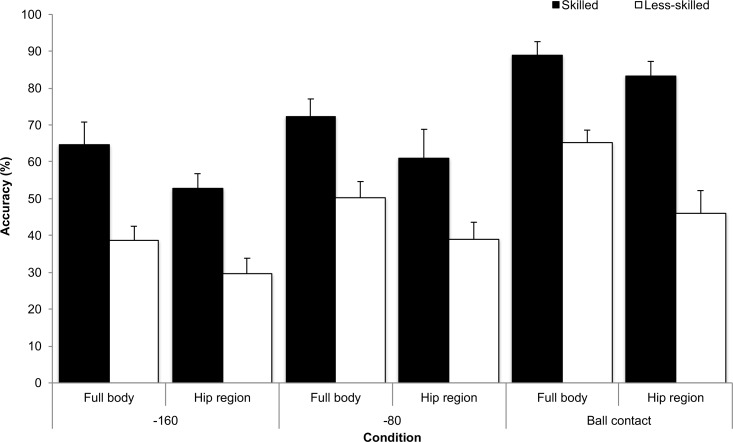
Correct response (%; SD) for the skilled and less-skilled groups in the full body and hip region spatial occlusion conditions and for the -160, -80 and ball contact temporal occlusion conditions.

There was a significant group x temporal occlusion interaction, *F*_2, 44_ = 6.50, *p* = 0.003, partial eta squared = 0.23. Both groups improved their response accuracy from the earliest occlusion point to the latest. However, while the less-skilled group increased performance incrementally by approximately 10% in each condition, the skilled group demonstrated a larger increase from -80 ms to ball contact (mean difference (MD) = 19%). There was a significant group x spatial occlusion interaction, *F*_1, 22_ = 6.66, *p* = 0.017, partial eta squared = 0.23, see Fig 2. Both skill groups performed significantly better in the full body condition, compared to the hip region condition. However, the skilled group managed to maintain performance to a better extent (MD = 10%) in the hip region condition, compared to the less-skilled group (MD = 13%).

There was a significant group x temporal occlusion x spatial occlusion interaction, *F*_2, 44_ = 11.63, *p* < 0.001, partial eta squared = 0.35, see Fig 2. Both groups performed better in the later occlusion points, compared to earlier occlusion points, and in the full body condition, compared to hip region condition. However, the skilled group’s response accuracy in the ball contact condition did not significantly differ between the full body and hip region condition (MD = 6%), whereas the less-skilled group showed a significant decrease in accuracy (MD = 19%). No other interactions were significant. All t-tests against chance were significant (all *p* < .05).

### Correct direction (%)

There was a significant main effect for group, *F*_1, 22_ = 45.988, *p* < 0.001, partial eta squared = 0.68. The skilled group recorded significantly higher accuracy scores (*M* = 85, *SD =* 11%), compared to the less-skilled group (*M* = 67, *SD =* 14%). There was a significant main effect for temporal occlusion, *F*_2, 44_ = 56.866, *p* < 0.001, partial eta squared = 0.72. Response accuracy was significantly higher in the ball contact (*M* = 80, *SD =* 16%) when compared to the -80 ms (*M* = 78, *SD =* 15%; *p* < 0.001) and -160 ms (*M* = 70, *SD =* 14%; *p* < 0.001) conditions. Response accuracy was also significantly higher in the -80 ms when compared to the -160 ms condition (*p* < 0.001). There was a significant main effect for spatial occlusion, *F*_1, 22_ = 27.555, *p* < 0.001, partial eta squared = 0.56. Response accuracy was significantly higher in the full body condition (*M* = 82, *SD =* 12%; *p* < 0.001) compared to the hips only condition (*M* = 69, *SD =* 16%).

**Fig 3:**
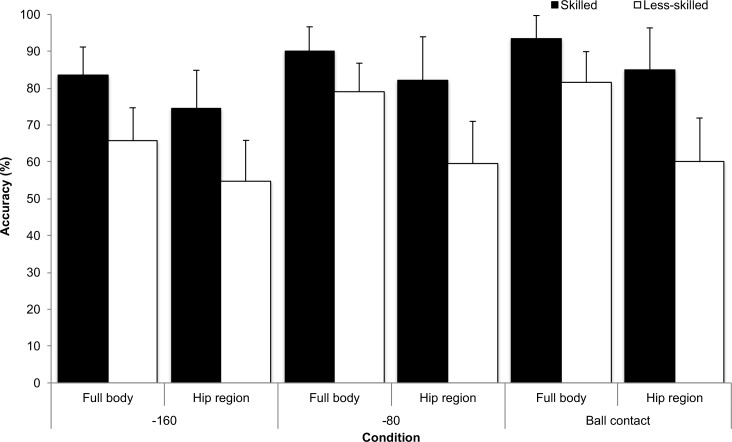
Correct direction (%; SD) for the skilled and less-skilled groups in the full body and hip region spatial occlusion conditions and for the -160, -80 and ball contact temporal occlusion conditions.

There was a significant group x temporal occlusion x spatial occlusion interaction, *F*_2, 44_ = 5.567, *p* = 0.007, partial eta squared = 0.20, see Fig 3. Both groups performed better in the later occlusion points, compared to earlier occlusion points, and in the full body condition, compared to hips only condition. However, the performance decrement from full body to hip region for the skilled group’s response accuracy in the ball contact condition was 9%, compared to 22% for the less-skilled group. Furthermore, there was no significant difference in response accuracy in the hip region condition for the less-skilled group across the three temporal occlusion conditions (*p* < 0.05). No other interactions were significant. For the less-skilled group in the -160 ms hip region conditions performance scores were not significantly different to chance (*t*_22_ = 1.514, *p* = 0.14). All other t-tests were significant (all *p* < .05).

### Correct height (%)

**Fig 4:**
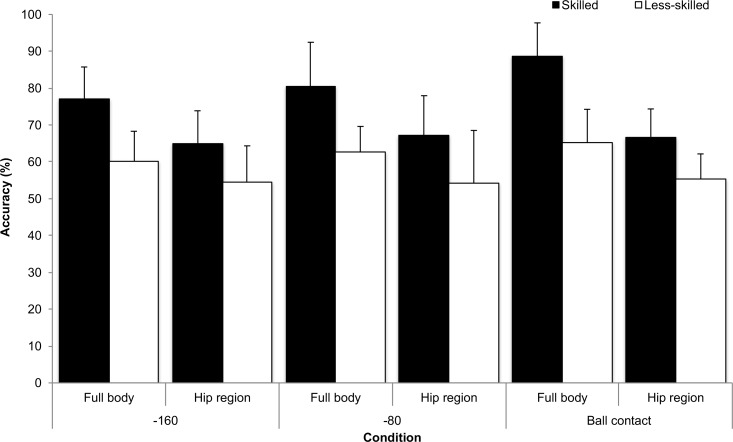
Correct height (%; SD) for the skilled and less-skilled groups in the full body and hip region spatial occlusion conditions and for the -160, -80 and ball contact temporal occlusion conditions.

There was a significant main effect for group, *F*_1, 22_ = 77.429, *p* < 0.001, partial eta squared = 0.78. The skilled group recorded significantly higher accuracy scores (*M* = 74, *SD =* 13%) when compared to the less-skilled group (*M* = 59, *SD =* 10%). There was a significant main effect for temporal occlusion, *F*_2, 44_ = 2.919, *p* = 0.045, partial eta squared = 0.15. Follow up analysis showed that response accuracy was significantly higher in the ball contact (*M* = 69, *SD =* 15%) when compared to the -160 ms (*M* = 64, *SD =* 12%; *p* = 0.017) condition. There was a significant main effect for spatial occlusion, *F*_1, 22_ = 41.118, *p* < 0.001, partial eta squared = 0.65. Response accuracy was significantly higher in the full body condition (*M* = 72, *SD =* 14%) when compared to the hip region condition (*M* = 61, *SD =* 11%). There was a significant group x spatial occlusion interaction, *F*_1, 22_ = 4.499, *p* = 0.045, partial eta squared = 0.17. The performance decrement between the full body and hip region conditions for the less-skilled group was significantly smaller (MD = 8%) relative to the skilled group (MD = 16%), see Fig 4. No other interactions were significant. For the less-skilled group in the -160 ms (*t*_22_ = 1.639, *p* = 0.12) and -80 ms (*t*_22_ = 1.045, *p* = 0.31) hip region conditions performance scores were not significantly different to chance. All other t-tests were significant (all *p* < .05).

## Discussion

Our aim in this study was to examine the effect of expertise on the accuracy of anticipatory judgments during temporally and spatially occluded soccer penalty kicks. It was hypothesized that there would be significant performance decrements for both groups in the spatially occluded condition, compared to the full body condition [21]. It was also predicted that the skilled group would perform significantly better than the less-skilled group in all conditions [22]. Finally, we predicted that both groups would perform significantly better in the later temporal occlusion conditions, compared to the earlier conditions [20, 23].

In support of the main hypotheses, the skilled group outperformed the less-skilled group on all three dependent variables, which supports a plethora of previous research [22]. Overall, participants performed better in the full body when compared the condition where only the hip region was presented. This finding supports our predictions and previous literature demonstrating that access to more information/cues can enhance performance [21]. There was partial support for the temporal occlusion prediction and previous research [7], as later occlusion conditions were associated with increased performance in the correct response, correct direction and correct height judgement categories. However, this effect was less pronounced in the correct height analysis, with no differences evident between the -160ms and -80ms conditions. These data indicate that critical cues for determining ball height may not be available until late in the moment, or as suggested in previous research, until the first portion of ball flight is visible [15].

When taken together, the spatial and temporal occlusion data suggest that skilled goalkeepers can use information from the hip region to accurately anticipate penalty-kick direction. In all occlusion conditions, the skilled goalkeepers reported accuracy scores significantly above chance level. In contrast, less-skilled players are less able to use this isolated area to make accurate judgments, albeit it should be noted that this group also reported accuracy scores above chance level. However, this finding does not necessarily mean that the hip region is critical to anticipation performance, or that this is necessarily the cue that the skilled goalkeepers use, as performers are able to extract information from several different sources. For example, experts are able to use other sources of information when the primary source is not available, which demonstrates their ability to adapt to task constraints in a given situation in order to maintain performance. This suggestion is supported by evidence to suggest that experts can extract information simultaneously from different areas of the body using a more global rather than local perceptual strategy [24, 25].

All participants were less accurate in predicting height when compared with side across all the spatial and temporal occlusion conditions, suggesting that there are limited cues available to predict height until after ball contact. In fact, the less-skilled participants were no better than chance at predicting height in the -80 ms and -160 ms hip region conditions, which suggests they are unable to extract any critical cues for height anticipation from this area, unlike the skilled players. This finding supports previous research suggesting, that the upper body, non-kicking foot, and initial ball flight are critical cues for successfully anticipating the height of the ball [15]. This finding would also explain why both groups were more successful at anticipating height when these cues were present in the full body condition.

In terms of anticipating direction, the skilled group was significantly more accurate compared to the less-skilled group. Specifically, the skilled group were less influenced by occlusion at ball contact, suggesting that the hips may contain useful cues at this time point to predict direction. Conversely, the less-skilled group’s anticipation accuracy was lower in the hip only condition across all temporal occlusion conditions, suggesting that they cannot use cues from the hips to predict direction, even at ball-contact. Specifically, in the -160 ms hip region condition, the less-skilled players were no better than chance at predicting direction, suggesting that they are unable to extract the necessary information from the hip region for successful anticipation. These data suggest that experts are utilizing information from the hip region, rather than simply using the area as a ‘visual pivot’, or ‘anchor point’ as suggested previously [11]. Previously, researchers have suggested that it is the relationships, presumably the relative motions between body segments and the ball, that are important when attempting to anticipate an opponent’s intentions [26, 27]. However, data from the current study suggests that expert athletes are able to use a single source of information, when needed, to infer upcoming opponent actions, albeit the players could have used the relative motion between the hip region and the ball. Nevertheless, this does not imply that this is the most effective or typical method of cue utilisation.

Moreover, under normal task constraints, skilled performers pick up information in a more distributed, global and continuous manner rather than relying exclusively on the serial processing of a single or local source of information [24, 25, 28]. Therefore, because experts are not overly reliant on one source of information, when only limited information is available, the skilled players would still be able to maintain anticipation accuracy. This adaptability is likely developed over extensive deliberate practice through the development of domain-specific knowledge and an ability to use postural cue information to predict future actions [29].

The temporal occlusion data in the current study corroborates previous research showing how the importance of different cues evolves across the movement [20, 23]. The traditional and robust temporal occlusion effect was present, with an increase in accuracy at the later occlusion points [7]. However, it appears that at -80 before ball contact skilled athletes are able to demonstrate extremely high anticipation accuracy for direction judgements. This finding seems to be a critical time point for direction prediction, as significantly decreases in performance have been noted when information in the hip region has been disguised [20]. Furthermore, the angle of the hips has been showed to correlate to ball direction between 150 and 50 ms before ball contact [18]. This finding would suggest that the relative importance of different cues alter throughout an action, with the accumulation of information from each of these cues critical for successful anticipatory judgments.

Although the players used to create the test film were asked to execute their penalty kicks as they would in a normal competition environment, this meant that deception and disguise of movements were not controlled, which has been shown to influence anticipation accuracy [23]. Furthermore, adding a competitive task during filming may have enhanced motivation of the players when taking the kicks and enhance the validity of the trials.

In summary, the current study demonstrates the expert advantage in anticipation performance. However, it appears that despite experts typically using a global strategy to pick up cues, they are able to adapt to pick up information from a single, local region to make highly accurate predictions, significantly above chance level. Furthermore, it appears that there is enough information within the hip region to make highly accurate predictions of penalty kick direction, however, other information is needed in order to make predictions of height. These data have implications for anticipation training, as well as providing a base for further research isolating critical cues, and the evolution of cues over time as an action unfolds.

## Supporting information

S1 File Mean Correct response (%; SD), correct direction (%; SD) and correct height (%; SD) data for the skilled and less-skilled groups in the full body and hip region spatial occlusion conditions and for the -160, -80 and ball contact temporal occlusion conditions. (XLSX)
